# Supplementary material for: Histone demethylase JMJD1C is phosphorylated by mTOR to activate de novo lipogenesis
Source: Nat Commun. 2020 Feb 7;11:796. doi: 10.1038/s41467-020-14617-1 (PMC7005700; doi:10.1038/s41467-020-14617-1)
Supplement: Supplementary file 1 — Supplementary Information [file 41467_2020_14617_MOESM1_ESM.pdf]

Histone demethylase JMJD1C is phosphorylated by mTOR to activate de novo lipogenesis

Viscarra et al.

PCR primers used

Mouse	Human
qPCR	
Fas	FAS
caagtgtccaccaacaagcg	aacggcaacctggtagtgag
ggagcgcaggatagactcac	gtgtccatgaagctcaccca
Srebf1	SREBF1
cagactcactgctgctgaca	gtttccgaggaacttttcgccg
cctccactcaccagggtct	cttcaccttcgatgtcggtca
Acc1	ACC
ttcactgtggcttctccagc	gccgcttgcctgacttttg
atcgcatgcatttcactgct	gtctggttcatccacgagca
Acly	ACLY
ttcctccttaatgccagcgg	cggacttcggcagaggtaga
agggatcttggacttgggact	ggagttctttgcccgtctgc
Acox1	ACOX
gcggtcccttgaccttttacctt	cctcggtcggtgcttacttc
gaagcaaggtgggcaggaaca	tcgggaaaggagggaggtc
Acaa1	ACAA
gagagcgctggcaaatcggtg	tgtttagggtgttggcggag
ggcggacaccctcatcctga	gggctcggatagaccacaac
Gapdh	GAPDH
ggacactgagcaagagaggc	gaaagcctgccggtgactaa
ttatgggggtctgggatgga	ttcccgttctcagccttgac
Gpam	GPAM
cggtgcttctctggggttac	gaagctggagctgctaggg
agggctttgcttactggtcc	ccacactcaccccattcctc
Scd1	SCD
cgcccctacgacaagaacat	aggcagcctcctttgtgtgt
ctcagaagcccaaagctcag	agggtttgccagccttgtct
Pgc1a	
tctcagtaaggggctggttg	
ttccgattggtcgctacacc	

G6pc
cacatccggggcatctacaa
caggtagaatccaagcgcga

Pck1
tgaaaggccgcaccatgtat
gcacagatatgcccatccga

Gk
tggcctaatgaaagctgggg
tagccacggaaccttccaac

ChIP

Fas promoter
agccccgacgctcattgg
cgctatttaaaccgcggcca

Acc1 promoter
cagtgttccgtacaggtgct
acagcatctcacagtcaggc

Srebf1 promoter
ccctccctggttgactcgta
gaggttcttcggcgtggatt

Acox1 promoter
acgacaatgaaccgtctcccc
ggactcacctcctttcaggc

Gpam promoter
ctgcagtccttgcctcttca
ggtctgtctgtctgtctgcc

FAS promoter
cagccccgacgctcattggc
gccggcgccgacgctattta

ACC promoter
cgaggcttgttgggcttcta
gtcagtcaccatcatggccc

SREBF1 promoter
gatgtaactggcccagcagt
tgtttctccagcaccaagca

ACOX promoter
ccaacctgttctaggcgtcg
gtcaagaaactgccctgctg

Supplementary Figure 1

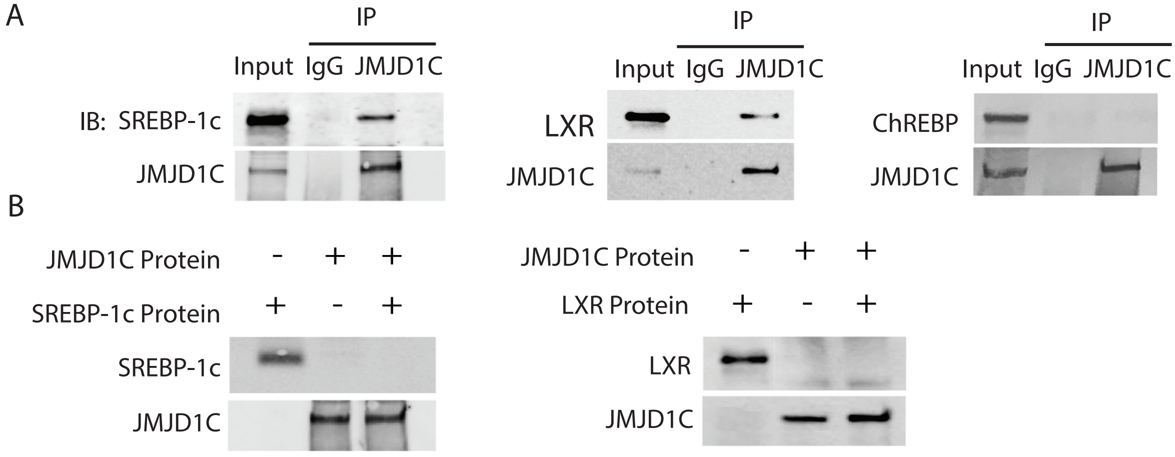

Supplementary Figure 1. A) Immunoblotting after co-IP of cell lysates of HEK293 cells co-transfected with JMJD1C and SREBP-1c, LXR or ChREBP after IP with JMJD1C antibody. B) Immunoblotting after in vitro incubation of purified JMJD1C with purified SREBP-1c or LXR. n=3 separate IP reactions.

Supplementary Figure 2

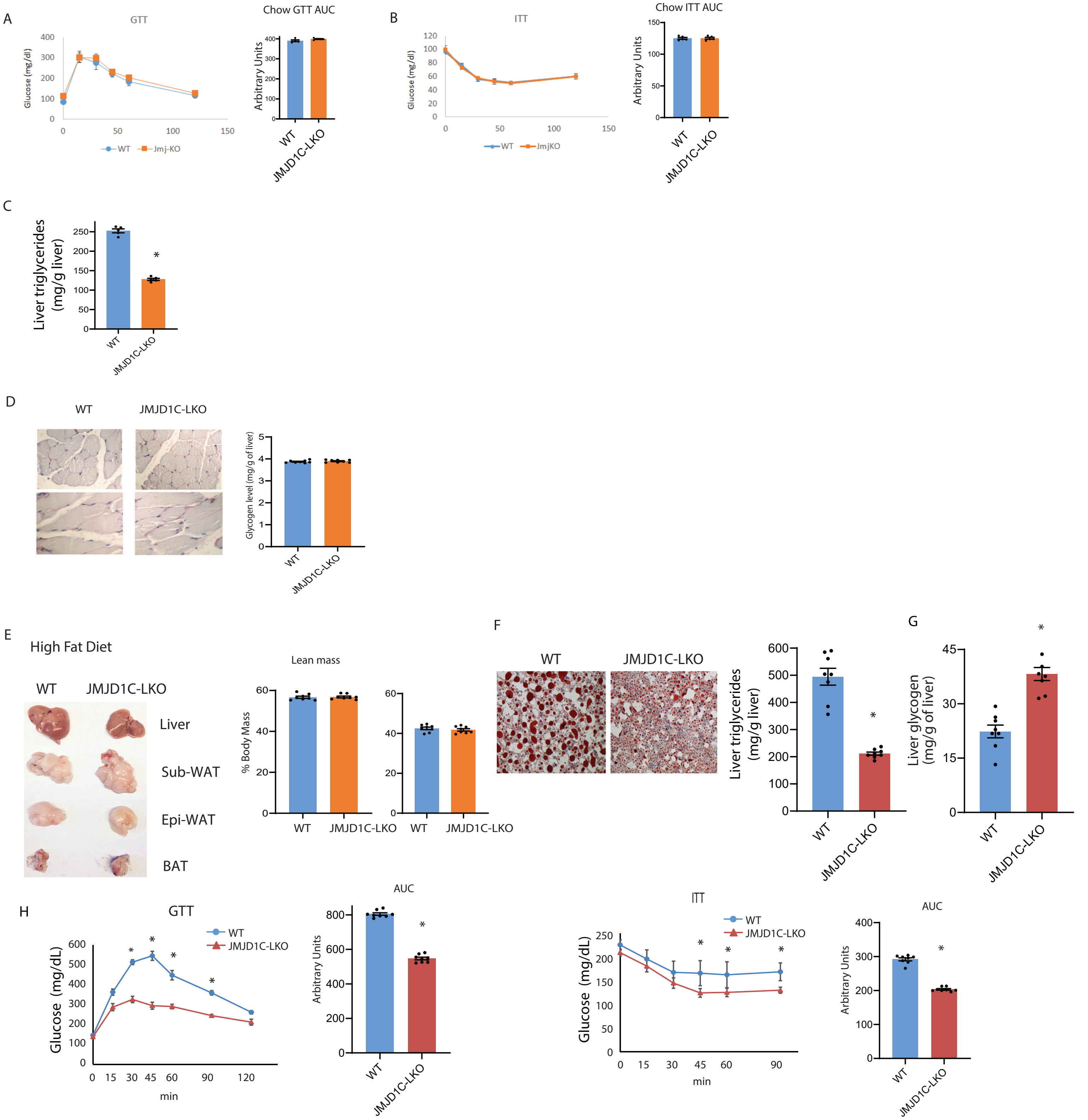

Supplementary Figure 2. A)GTT and B) ITT and AUC analysis of mice maintained on chow diet for 4 months, n=5. C) Liver triglyceride levels of WT and JMJD1C-LKO mice maintained on chow diet for 4 months, n=5. D) Glycogen staining and quantification in muscle of WT and JMJD1C-LKO mice. (E-H) JMJD1C-LKO mice and WT littermates were fed a high fat diet (45% kcal% fat) for 3 months after weaning. E left) Pictures shown are liver, subcutaneous white adipose (Sub-WAT), epididymal white adipose (Epi-WAT), and brown fat. (E right) MRI analysis of body composition. F) Oil red O staining of liver tissue sections. G) liver glycogen levels. H) GTT and ITT for mice on HFD and AUC analysis. n=8 per groups. Data are expressed as means ±SEM. *P < 0.05, **P < 0.01.

Supplementary Figure 3

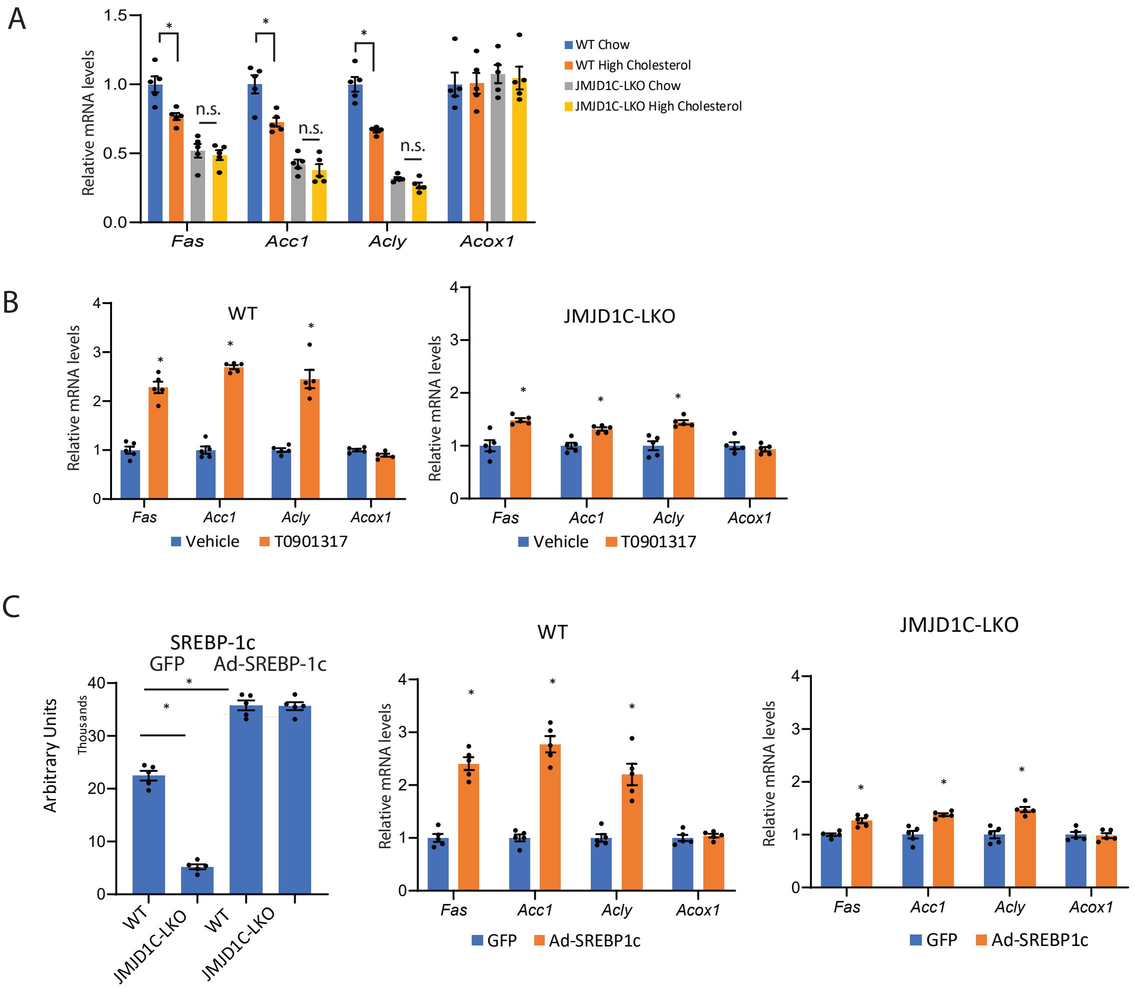

Supplementary Figure 3. A. WT and JMJD1C-LKO mice were maintained on a high cholesterol diet (HChD) for 8 weeks. RT-qPCR for lipogenic genes using RNA extracted from livers of these mice. B. WT and JMJD1C-LKO mice were IP injected with either vehicle, DMSO or LXR agonist T0901317 (50mg/kg) daily for 5 days. RT-qPCR for lipogenic genes using RNA extracted from livers of these mice. C. WT and JMJD1C-LKO mice were injected with adenovirus for SREBP-1c via tail vein. After 10 days, livers were harvested and mRNA extracted for RT-qPCR for lipogenic genes. A-C) n=5 for all, * denotes $p<0.05$. Data are expressed as means ±SEM.

Supplementary Figure 4

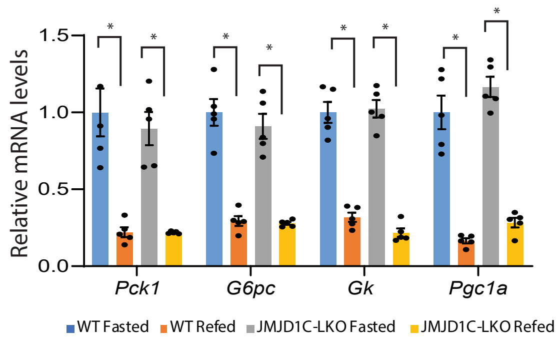

Supplementary Figure 4. WT and JMJD1C-LKO mice were fasted overnight then refed with high CHO diet for 6 hrs. RT-qPCR for gluconeogenic genes using RNA extracted from livers of these mice shows no difference in response to feeding between WT and JMJD1C-LKO. n=5 for all, * denotes $p<0.05$. Data are expressed as means ±SEM.

Supplementary Figure 5

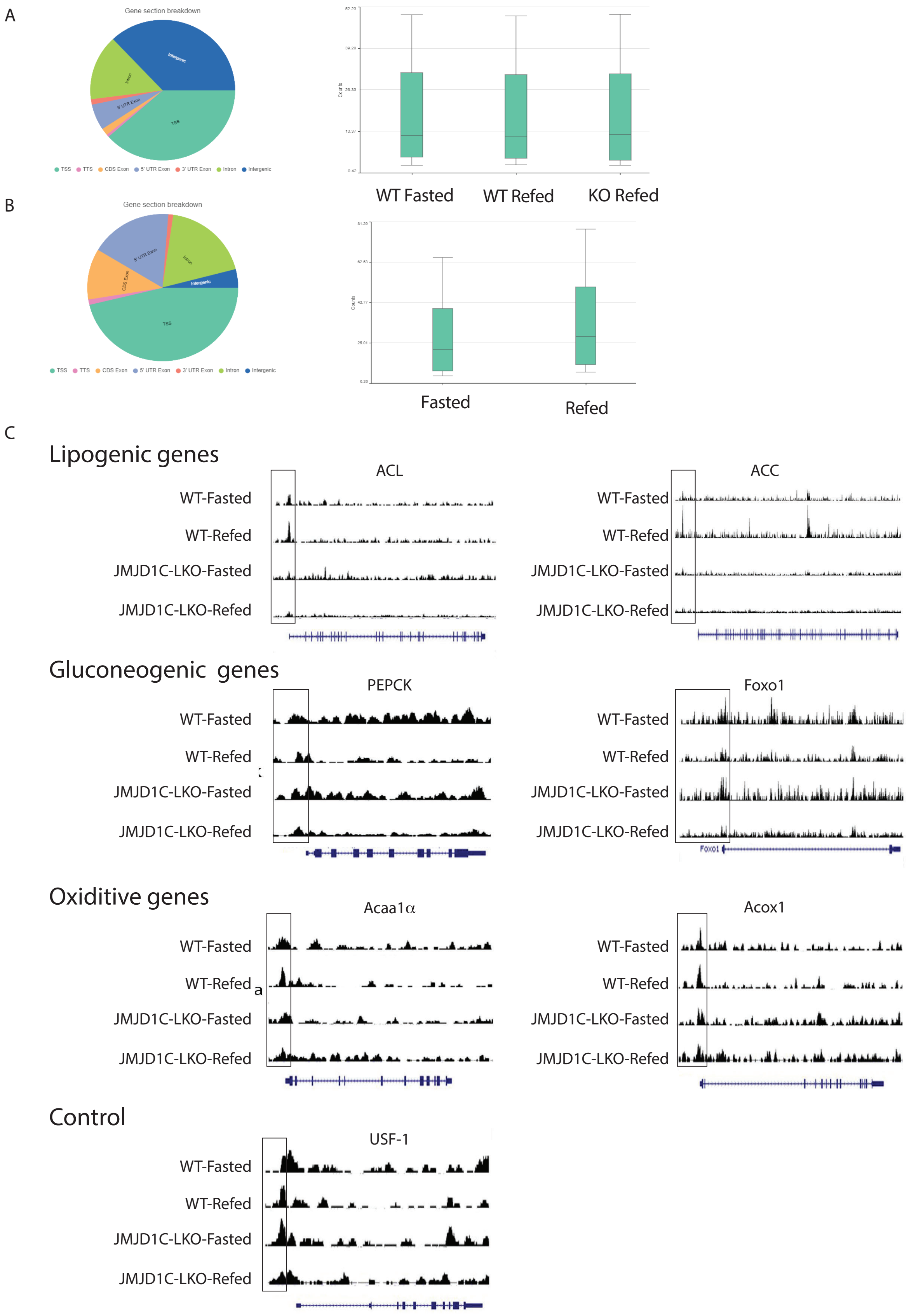

Supplementary FIgure 5. A and B) Gene section breakdown showing where peaks were found genome wide for ATAC- and ChIP-seq results and box plots showing number and distribution of reads between groups. C) ATAC-seq results show promoter regions arer more open in refed stage compared to fasted stage in WT lipogenic genes. These regions are closed in JMJD1C-LKO both in fasted and refed stages. While promoter regions of gluconeogenic and oxidative genes are more open in fasted stage than refed stage. Promoter region of USF-1 is shown as control as it is not regulated by fasting/feeding. Peak shots for each gene shown were taken at the same height so that peak heights could be compared. n=2 biological replicates.

Supplementary Figure 6

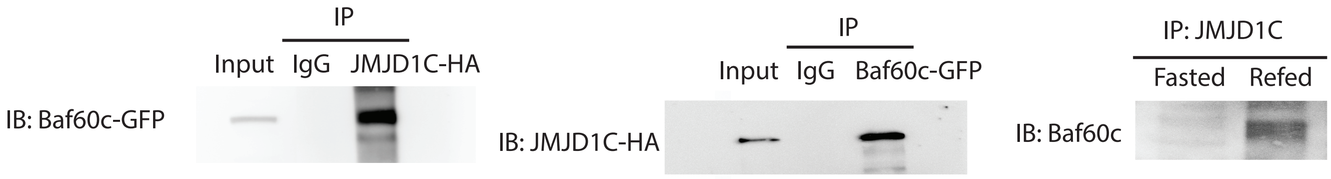

Supplementary Figure 6. Immunoblotting with GFP antibody after co-IP of cell lysates of HEK293 cells co-transfected with JMJD1C-HA and Baf60c-GFP using HA antibody (left) and immunoblotting with HA antibody after co-IP with GFP antibody (middle). Immunoblotting with Baf60c antibody after co-IP of liver lysates from fasted and fed mice using JMJD1C antibody (right). Results showed the interaction between JMJD1C and Baf60c in cells and endogenous JMJD1C interaction with Baf60c in mouse liver extract in fed but not fasted mice. n=3 independent IP reactions.

Supplementary Table 1

category	protein	peptide sequence
a) DNA break/repair components	DNA-PK	R.CGAALAGHQLIR.G 2/ R.ICSKPVVLPK.G2
		R.LYSLALHPNAFKR.L2/ K.WLLAHCGRPQTECR.H2
		R.FNNYVDCMKK.F2 /K.INQVFHGSCITEGNELTK.T2
		R.SSFDWLTGSSTDPLVDHTSPSSDSLLFAHK.R3
		R.LGLPGDEVDNKVK.G2 /R.LLQIIERYPEETLSLMTK.E2
		K.GANRTETVTSFR.K 2 /K.KGGSWIQEINVAEKNWYPR.Q3
		K.KGGSWIQEINVAEKNWYPR.Q2
	Ku70	R.ILELDQFKGQQGQKR.F2 / R.IMLFTNEDNPHGNDSAK.A2
		K.AGDLRDTGIFLDLMHLK.K2 / K.TRTFNTSTGGLLLPSDTKR.S3
		K.TRTFNTSTGGLLLPSDTKR.S2 /R.TFNTSTGGLLLPSDTKR.S2
		K.CLEKEVAALCR.Y2 /R.NLEALALDLMEPEQAVDLTLPK.V3
		R.NLEALALDLMEPEQAVDLTLPKVEAMNK.R3
		R.NLEALALDLMEPEQAVDLTLPKVEAMNKR.L3 /K.GTLGKFTVPMLK.E 2
		K.SGLKKQELLEALTK.H/R.LGSLVDEFKELVYPPDYNPEGK.V2
	ku80	R.HLMLPDFDLLEDIESK.I2 /K.KYAPTEAQLNAVDALIDSMSLAK.K2
		K.YAPTEAQLNAVDALIDSMSLAK.K2 /R.LFQCLLHR.A2
		K.IKTLFPLIEAK.K2/K.ASFEEASNQLINHIEQFLDTNETPYFMK.S
	PARP-1	K.CSESIPKDSLR.M2 /K.TAEAGGVTGKGQDGIGSKAEK.T2
		K.RKGDEVDGVDEVAK.K2 /K.VCSTNDLKELLIFNK.Q2
		R.VVSEDFLQDVSASTK.S2 /K.SKLPKPVQDLIK.M2
		K.KPPLLNNADSVQAK.V
	Topoisomerase	K.GIPVVEHKVEK.V2/ R.RLHGLPEQFLYGTATK.H2
		R.LHGLPEQFLYGTATK.H2
b) protein phosphatase	PP1	K.NVQLQENEIR.G2 /K.IKYPENFFLLR.G2
		K.IFCCHGGLSPDLQSMEQIRR.I2 /K.IFCCHGGLSPDLQSMEQIRR.I3
		K.TFTDCFNCLPIAAIVDEK.I2/K.YGQFSGLNPGGRPITPPR.N2
		K.TFTDCFNCLPIAAIVDEK.I/K.YGQFSGLNPGGROITOOR.N
c) Histone modification proteins	P/CAF	K.MTDSHVLEEAKKPR.V2/K.MTDSHVLEEAK#KPR.V2
		K.HDILNFLTYADEYAIGYFK.K2 /K.HDILNFLTYADEYAIGYFKK.Q2
		K.YVGYIKDYEGATLMGCELNPR.I2 /K.SK#EPRDPDQLYSTLK.S2
		K.SHQSAWPFMEPVKR.T2 /K.SHQSAWPFMEPVKR.T3
		R.VFTNCKEYNPPESEYYK.C2
	HDAC9	K.QLQQELLLIQQQQQIQK.Q2
	JMJD1C	L.RSVASTSA.K/S.SSSSPKSHI.I
d) transcription factor	SREPBP	R.RQWTSLSLR.V/R.LRSPPEALVQGR.Y
		R.LKMMEEKGEPTQTK/K.SEVQQPVHPKPLSPDSR.A
		R.LLANQQVFHISCFR.C/K.LSLGTYASLHGR.I
		R.IYCKPHFNQLFK.S/R.IAWPPPTELGSSGSALEEGIK.M
		R.SRPFTVAASPQSTSVK.S
	SPTF-associated factor 65 gamma transtript	F.EEPMSGMS.E/E.EPMSGMS.E
	CCAAT/enhancer binding protein zeta	d.KTLESMSS.Y/L.GDRMAAM.I
	TTF-1 interacting peptide 5	D.KTLESMSS.Y
	trans-acting transcription factor 9	S.GAPQAS
	zinc finer, FYVE domain containing protein	T.S#TYQSLQDLVLPTPPYLFGIL.I
	Max-interacting protein	K.KALPS#KDK.A/P.RIGT#QLEGS#.S

Supplementary Table 2. Differentially enriched motifs between WT and JMJD1C-LKO datasets identified by ATAC-seq

Motif name	Consensus sequence	WT p-value	KO p-value
ASCL1 (MA1100.1)	NNNGCAGCTGBNN	4.54E-18	0.462776975
ATF7 (MA0834.1)	NNRTGACGTCAYNN	6.01E-05	0.227341629
BACH2 (MA1101.1)	NVTGASTCAGCANN	0.018313907	0.300210442
CREB1 (MA0018.3)	NNTGACGTCANN	1.70E-15	0.892847419
Creb5 (MA0840.1)	NATKACGTMAYN	0.004728147	0.977973064
Dux (MA0611.1)	NCAATCAD	4.22E-09	1
DUX4 (MA0468.1)	WAAHBBAATCA	1.24E-06	0.943651389
DUXA (MA0884.1)	NHRANBYAATCAN	1.08E-08	0.900459587
E2F4 (MA0470.1)	NSGCGGGARVN	2.43E-64	0.245882054
ELK4 (MA0076.2)	NNRYTTCCKSN	5.54E-18	0.380681719
FOSL2::JUND(var.2) (MA1145.1)	NNRTKACGTCAYNNN	4.02E-05	0.443577631
FOXC2 (MA0846.1)	WRNRYMAAYAWN	0.008238219	0.698890944
FOXD2 (MA0847.1)	RHAAAYA	3.39E-44	0.444716669
Foxj2 (MA0614.1)	RTMAACAN	2.47E-12	0.99999172
Foxj3 (MA0851.1)	NNNNNNRTAAAYAAWNNN	3.68E-07	0.212954586
FOXK1 (MA0852.2)	NNNGTAAACANNNN	0.000134403	0.915911583
FOXK2 (MA1103.1)	NNRTAAACANN	0.003692953	0.968388858
FOXL1 (MA0033.2)	RTAAAYA	2.18E-81	0.069345947
FOXO4 (MA0848.1)	RTMAAYA	5.46E-19	0.999824051
FOXP2 (MA0593.1)	NDRTAAACAVN	1.74E-19	0.184938386
FOXP3 (MA0850.1)	RYAARBA	5.86E-43	0.111048255
HNF1A (MA0046.2)	NRTHAATNATTAACN	1.43E-20	0.147930202
HNF1B (MA0153.2)	GTWAATNATTAAY	7.17E-13	0.526387781
HNF4G (MA0484.1)	NNRGDNCARAGKBCW	1.33E-06	0.065048916
HOXA10 (MA0899.1)	NNYMATWAANN	1.65E-05	0.853184413
HOXB13 (MA0901.1)	SYHATAAAMH	7.35E-19	0.126515447
HOXD13 (MA0909.1)	SYMATAAAAN	1.01E-99	0.142908847
HSF1 (MA0486.2)	TTCBVGAABNTTC	0.016436792	0.693558922
JDP2(var.2) (MA0656.1)	NATGACGTCAYN	3.11E-06	0.163048184
KLF14 (MA0740.1)	NRHCACGCCCMYHN	7.86E-52	0.087368345
MEF2A (MA0052.3)	DCTAWAAATAGM	0.028949313	0.664227117
MEF2D (MA0773.1)	DCTAWAAATAGM	0.030188327	0.539837991
Myog (MA0500.1)	VRCAGSTGNNN	4.72E-06	0.956684815
NFIX (MA0671.1)	NNNGCCARN	0.004788364	0.994481932
NKX2-3 (MA0672.1)	NVCACTTVDV	7.02E-09	0.362523681
NKX2-8 (MA0673.1)	VYACTYVWN	3.20E-39	0.627598177
Nkx3-1 (MA0124.2)	NVCACTTAN	3.97E-11	0.996912604
NR2F2 (MA1111.1)	NNAAGGTCANN	0.051074687	0.875078749
PBX2 (MA1113.1)	NDGABTGACANN	1.74E-29	0.074805253
PRDM1 (MA0508.2)	NNACTTTCHH	6.35E-33	0.998154352
REST (MA0138.2)	NYCAGBACCNHGGASAGN[illegible]	0.044245253	1
RFX2 (MA0600.2)	NGTTRCYATRGYAACN	9.51E-12	1
SOX10 (MA0442.2)	NNNACAAAGNN	3.98E-50	0.887332385
SOX13 (MA1120.1)	NNACAATGNNN	3.10E-06	0.86627787
Spz1 (MA0111.1)	RSRGTWDSRKV	2.56E-37	0.068879689
Stat6 (MA0520.1)	NNNTTCYDVDGAANN	6.31E-22	0.64424378
Tcf12 (MA0521.1)	VRCAGCTGNNN	0.000335686	0.891462616
Tcf7 (MA0769.1)	NDASATCAAAGN	0.003699129	0.658937589
TCF7L1 (MA1421.1)	HRASATCAAAGR	0.001286485	0.681851533
TFAP2A (MA0003.3)	HSCCBNVRGCN	0.000323375	0.151497173
TFDP1 (MA1122.1)	NVGCGGGAANN	4.50E-55	0.111260955
TGIF1 (MA0796.1)	TGACAGSTGTCA	0.048065388	1
USF (MA0526.2)	NNNNGTCACGTGRBNN	9.52E-08	0.771507598
Zfx (MA0146.2)	NNVNSYBVGGCCTN	2.15E-14	0.633917443
ZNF143 (MA0088.2)	YWCCCAYRATGCAHYR	0.01087617	1
