# Supplementary material for: Histone demethylase JMJD1C is phosphorylated by mTOR to activate de novo lipogenesis
Source: Nat Commun. 2020 Feb 7;11:796. doi: 10.1038/s41467-020-14617-1 (PMC7005700; doi:10.1038/s41467-020-14617-1)
Supplement: Supplementary file 3 — Reporting Summary [file 41467_2020_14617_MOESM3_ESM.pdf]

nature research

Corresponding author(s): Hei Sook Sul

Last updated by author(s): 01/07/2020

Reporting Summary

Nature Research wishes to improve the reproducibility of the work that we publish. This form provides structure for consistency and transparency in reporting. For further information on Nature Research policies, see Authors & Referees and the Editorial Policy Checklist.

Statistics

For all statistical analyses, confirm that the following items are present in the figure legend, table legend, main text, or Methods section.

n/a	Confirmed	
☐	☒	The exact sample size (n) for each experimental group/condition, given as a discrete number and unit of measurement
☐	☒	A statement on whether measurements were taken from distinct samples or whether the same sample was measured repeatedly
☐	☒	The statistical test(s) used AND whether they are one- or two-sided<br>*Only common tests should be described solely by name; describe more complex techniques in the Methods section.*
☐	☒	A description of all covariates tested
☐	☒	A description of any assumptions or corrections, such as tests of normality and adjustment for multiple comparisons
☐	☒	A full description of the statistical parameters including central tendency (e.g. means) or other basic estimates (e.g. regression coefficient) AND variation (e.g. standard deviation) or associated estimates of uncertainty (e.g. confidence intervals)
☒	☐	For null hypothesis testing, the test statistic (e.g. F, t, r) with confidence intervals, effect sizes, degrees of freedom and P value noted<br>*Give P values as exact values whenever suitable.*
☒	☐	For Bayesian analysis, information on the choice of priors and Markov chain Monte Carlo settings
☒	☐	For hierarchical and complex designs, identification of the appropriate level for tests and full reporting of outcomes
☒	☐	Estimates of effect sizes (e.g. Cohen's d, Pearson's r), indicating how they were calculated

Our web collection on statistics for biologists contains articles on many of the points above.

Software and code

Policy information about availability of computer code

Data collection	*Provide a description of all commercial, open source and custom code used to collect the data in this study, specifying the version used OR state that no software was used.*
Data analysis	Partek flow genomics suite was used in analysis of ChIP-, ATAC-, and RNA-seq data. GraphPad Prism 8 was used in analysis of all other data presented in the manuscript.

For manuscripts utilizing custom algorithms or software that are central to the research but not yet described in published literature, software must be made available to editors/reviewers. We strongly encourage code deposition in a community repository (e.g. GitHub). See the Nature Research guidelines for submitting code & software for further information.

Data

Policy information about availability of data

All manuscripts must include a data availability statement. This statement should provide the following information, where applicable:

- Accession codes, unique identifiers, or web links for publicly available datasets
- A list of figures that have associated raw data
- A description of any restrictions on data availability

The genomic and transcriptomic datasets generated and analyzed during the current study are available in the GEO repository under accession number: GSE142815[https://www.ncbi.nlm.nih.gov/geo/query/acc.cgi?acc=GSE142815].
The proteomic datasets generated and analyzed during the current study are available in the MassIVE repository under accession number MSV000084745[doi:10.25345/C53Q2F].

Field-specific reporting

Please select the one below that is the best fit for your research. If you are not sure, read the appropriate sections before making your selection.

☒ Life sciences ☐ Behavioural & social sciences ☐ Ecological, evolutionary & environmental sciences

For a reference copy of the document with all sections, see nature.com/documents/nr-reporting-summary-flat.pdf

Life sciences study design

All studies must disclose on these points even when the disclosure is negative.

Sample size	No sample size power calculations were performed.
Data exclusions	No data were excluded from analyses.
Replication	All experiments were repeated a minimum of 3 times to ensure reproducibility.
Randomization	Randomization was not employed.
Blinding	Blinding was not employed

Reporting for specific materials, systems and methods

We require information from authors about some types of materials, experimental systems and methods used in many studies. Here, indicate whether each material, system or method listed is relevant to your study. If you are not sure if a list item applies to your research, read the appropriate section before selecting a response.

Materials & experimental systems

n/a	Involved in the study	
☐	☒	Antibodies
☐	☒	Eukaryotic cell lines
☒	☐	Palaeontology
☐	☒	Animals and other organisms
☒	☐	Human research participants
☒	☐	Clinical data

Methods

n/a	Involved in the study	
☐	☒	ChIP-seq
☒	☐	Flow cytometry
☒	☐	MRI-based neuroimaging

Antibodies

Antibodies used	Rabbit polyclonal specific phospho-T505 of JMJD1C antibodies were raised against the peptide corresponding to aa 496-512 of JMJD1C (KEKFVSRPPTPKCVIDI) phosphorylated at T505 (P-T505) (Genemed). The specific antibody was affinity-purified using the phospho-peptide before use (diluted 1:500). The following commercial antibodies were used: JMJD1C (sc-101073, 1:500), GAPDH (sc-32233, 1:2000), USF-1 (sc-229, 1:1000), HDAC1 (sc-7872, 1:1000), Fatty acid synthase (sc-55580, 1:1000), and SREBP-1c (sc-366, 1:1000) from SCBT. FLAG (147935, 1:1000), HA (C29F4, 1:1000), and Raptor (24C12, 1:750) from CST. phospho-Threonine (05-1923, 1:200), phosphor-Serine (Ab1603, 1:200), and normal IgG (PP64B, 2ug/ug of chromatin) from EMD Millipore. GFP (ab1218, 1:1000), Total H3 (ab1791, 2ug/ug of chromatin), H3K9me1 (ab9045, 2ug/ug of chromatin), H3K9me2 (ab176882, 2ug/ug of chromatin), H3K9me3 (ab8898, 2ug/ug of chromatin), and H3K4me3 (ab8580, 2ug/ug of chromatin) from Abcam.
Validation	Commercial antibodies were validated by the supplier, we further validated antibodies through overexpression of tagged constructs to confirm specificity of the antibody. Our custom phospho-T505 antibody was validated through overexpression of both WT and our unphophorylatable T505A mutant JMJD1C to confirm its specificity.

Eukaryotic cell lines

Policy information about cell lines

Cell line source(s)	ATCC
Authentication	Cells were authenticated by the supplier.
Mycoplasma contamination	All cell lines tested negative for mycoplasma.

Commonly misidentified lines (See ICLAC register)	N/A

Animals and other organisms

Policy information about studies involving animals; ARRIVE guidelines recommended for reporting animal research

Laboratory animals	C57bl/6 mice were used beginning at 6 weeks of age.
Wild animals	*Provide details on animals observed in or captured in the field; report species, sex and age where possible. Describe how animals were caught and transported and what happened to captive animals after the study (if killed, explain why and describe method; if released, say where and when) OR state that the study did not involve wild animals.*
Field-collected samples	*For laboratory work with field-collected samples, describe all relevant parameters such as housing, maintenance, temperature, photoperiod and end-of-experiment protocol OR state that the study did not involve samples collected from the field.*
Ethics oversight	UC Berkeley Animal Care and Use Committee approved the study protocols used.

Note that full information on the approval of the study protocol must also be provided in the manuscript.

ChIP-seq

Data deposition

☒ Confirm that both raw and final processed data have been deposited in a public database such as GEO.

☒ Confirm that you have deposited or provided access to graph files (e.g. BED files) for the called peaks.

Data access links *May remain private before publication.*	[https://www.ncbi.nlm.nih.gov/geo/query/acc.cgi?acc=GSE142815]
Files in database submission	2 Fasted Input/ChIP, 2 Refed Input/ChIP, 2 Fasted ATAC, 2 Refed ATAC, 2 Refed JMJD1C-LKO ATAC, 2 Refed WT RNA, 2 Refed JMJD1C-LKO RNA. Raw and processed files for all.
Genome browser session (e.g. UCSC)	*Provide a link to an anonymized genome browser session for "Initial submission" and "Revised version" documents only, to enable peer review. Write "no longer applicable" for "Final submission" documents.*

Methodology

Replicates	For each seq experiment we have 2 biological replicates
Sequencing depth	Approximately 40 million, Single-end reads of 50bp
Antibodies	H3K9me2, Abcam, ab176882
Peak calling parameters	Described in more detail in methods, reads were aligned to mm10 genome assembly, MACS2 was used for peak calling
Data quality	*Describe the methods used to ensure data quality in full detail, including how many peaks are at FDR 5% and above 5-fold enrichment.*
Software	GSL provides standard compressed fastq files (demultiplexed when requested) as output by the Illumina supported CASAVA bcl2fastq2 (v2.20) program for data collection. Partek flow genomics suite was used for data analysis.
