# Supplementary material for: Histone demethylase JMJD1C is phosphorylated by mTOR to activate de novo lipogenesis
Source: Nat Commun. 2020 Feb 7;11:796. doi: 10.1038/s41467-020-14617-1 (PMC7005700; doi:10.1038/s41467-020-14617-1)
Supplement: Supplementary file 4 — Source Data [file 41467_2020_14617_MOESM4_ESM.zip › Combined Source Data.pdf]

Figure 1A

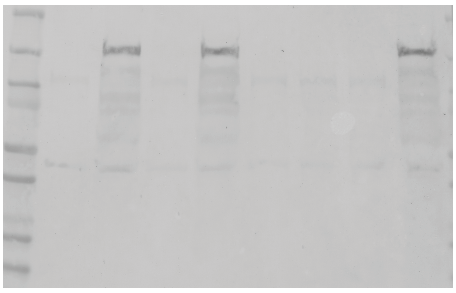

IB: FLAG-JMJD1C

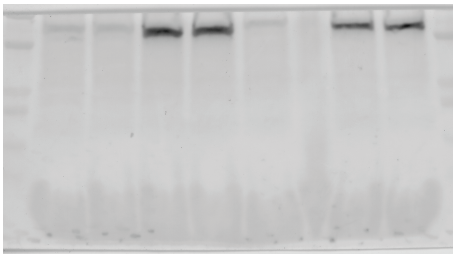

IB: HA-USF-1

Figure 1B

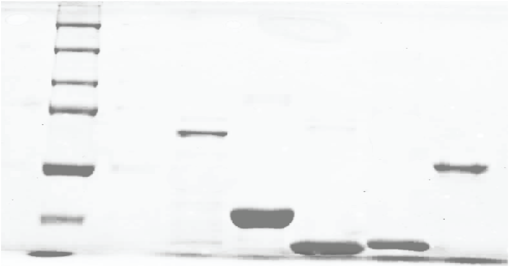

IB: GST-USF-1

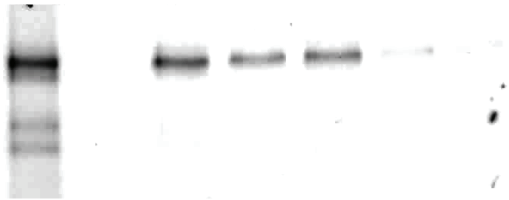

S^{35}-JMJD1C

Figure 1C

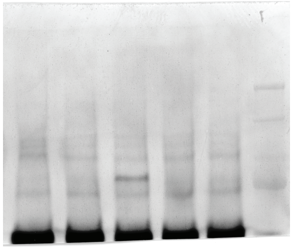

IB: FLAG-JMJD1C

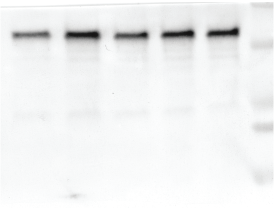

IB: HA-USF-1

Fig. 2A

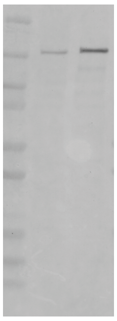
IB: JMJD1C

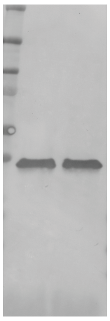
IB: GAPDH

Fig. 2B

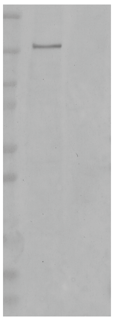
IB: JMJD1C

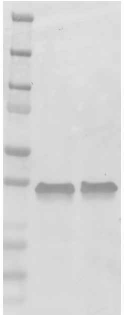
IB: GAPDH

Fig. 2C

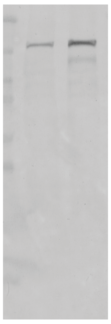
IB: JMJD1C

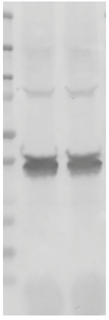
IB: GAPDH

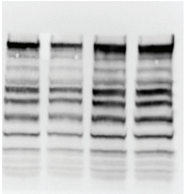
IB: FAS

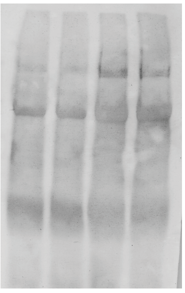
IB: SREBP-1c

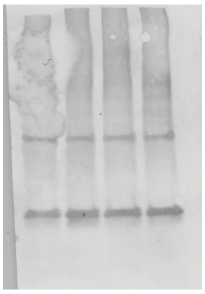
IB: GAPDH

Fig. 2D

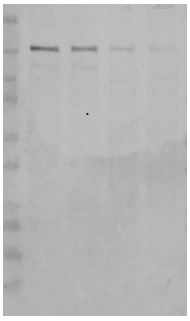
IB: JMJD1C

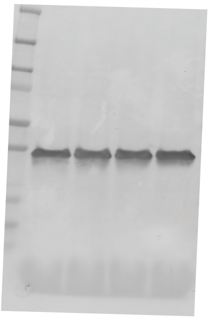
IB: GAPDH

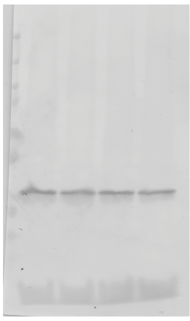
IB: GFP

Fig. 3A

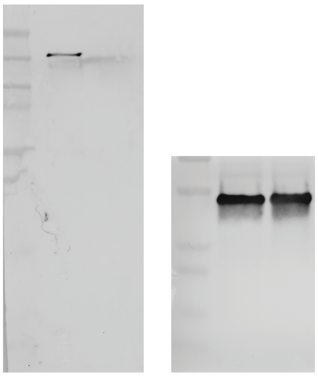

IB: JMJD1C IB: GAPDH

Fig. 3B

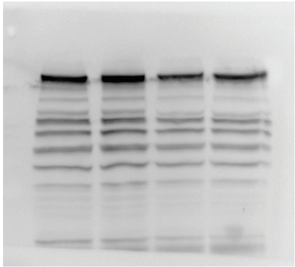

IB: FAS

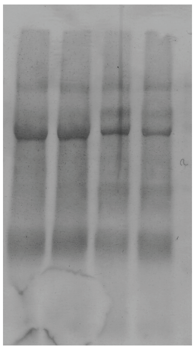

IB: SREBP-1c

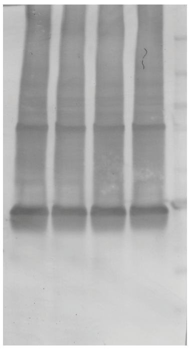

IB: GAPDH

Fig. 7B

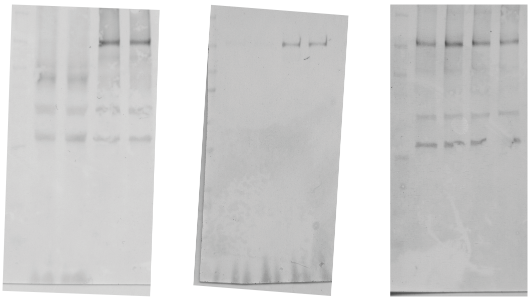

IB: p-Ser IB: p-Thr IB: JMJD1C

Fig. 7C

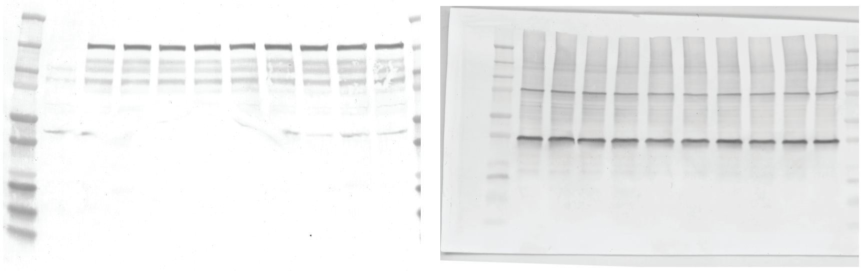

IB: FLAG-JMJD1C IB: GAPDH

Fig. 7D

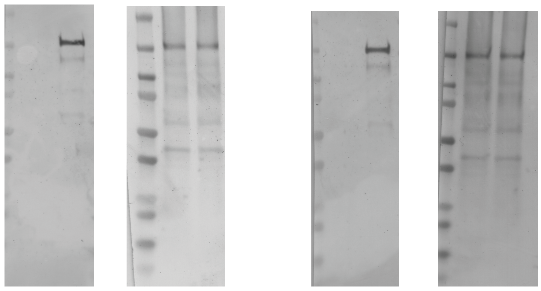

IB: p-T505 IB: JMJD1C IB: p-T505 IB: JMJD1C

Fig. 7E

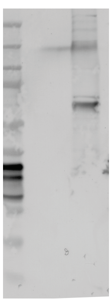

IB: p-Thr IB: GST-JMJD1C

Fig. 7E

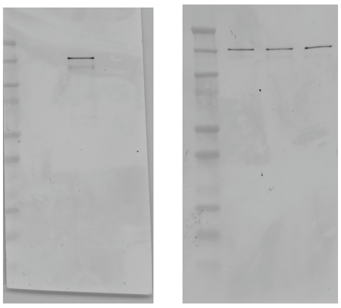

IB: p-T505 IB: JMJD1C

Fig. 7F

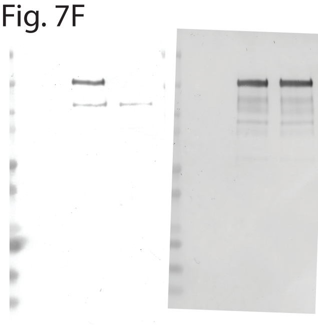

IB: p-T505 IB: JMJD1C

Fig. 7G

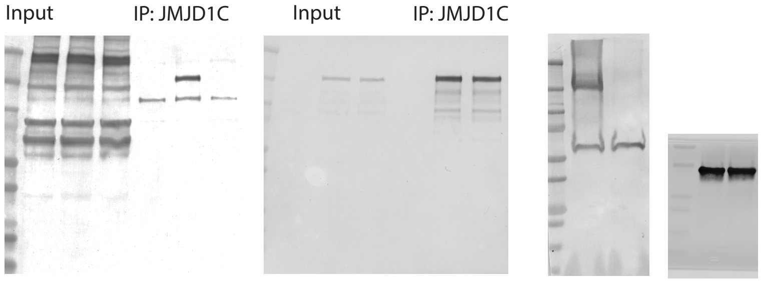

IB: p-T505 IB: JMJD1C IB: Raptor IB: GAPDH

Fig. 7H

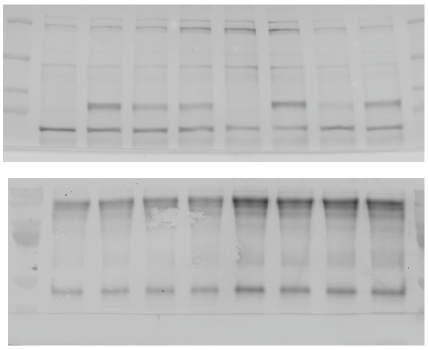

IB: FLAG-JMJD1C

IB: HA-USF-1

Fig. 7I

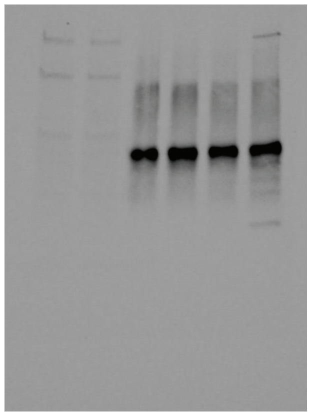

Fig. 7K

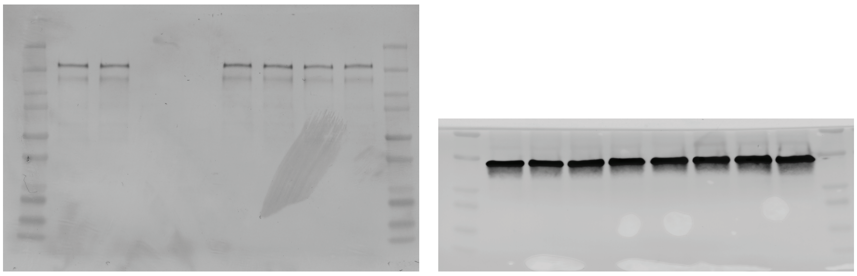

IB: JMJD1C IB: GAPDH
